# Spermatogonia apoptosis induction as a possible mechanism of *Toxoplasma gondii-*induced male infertility 

**DOI:** 10.22038/ijbms.2020.43535.10224

**Published:** 2020-09

**Authors:** Jasem Saki, Mohamad Sabaghan, Reza Arjmand, Ali Teimoori, Mohammad Rashno, Ghasem Saki, Saeedeh Shojaee

**Affiliations:** 1 Cellular and Molecular Research Center, Ahvaz Jundishapur University of Medical Sciences, Ahvaz, Iran; 2 Department of Parasitology, Faculty of Medicine, Ahvaz Jundishapur University of Medical Sciences, Ahvaz, Iran; 3 Virology Department, School of Medicine, Ahvaz Jundishapur University of Medical Sciences, Ahvaz, Iran; 4 Department of Immunology, School of Medicine, Ahvaz Jundishapur University of Medical Sciences, Ahvaz, Iran; 5 Physiology Research Center, School of Medicine, Ahvaz Jundishapur University of Medical Sciences, Ahvaz, Iran; 6 Department of Medical Parasitology and Mycology, School of Public Health, Tehran University of Medical Sciences, Tehran, Iran

**Keywords:** Apoptosis, Gene expression, In vitro, Spermatogonia, Toxoplasma gondii

## Abstract

**Objective(s):**

The protozoan *Toxoplasma gondii *as an intracellular protozoan is widely prevalent in humans and animals. Infection generally occurs through consuming food contaminated with oocysts and tissue cysts from undercooked meat. The parasite is carried in sexual fluids like semen but there is little information about the effect of *T. gondii* on the male reproductive system. In this study, we examined the effect of *T. gondii* tachyzoites on apoptosis induction in type B spermatogonia (GC-1) cells.

**Materials and Methods:**

Fresh tachyzoites taken of infected BALB/c mice, GC-1 spg cells were infected with increasing concentrations of tachyzoites of *T. gondii*, then apoptotic cells were identified and quantified by flow cytometry. The genes associated with apoptosis were evaluated by RT2 Profiler PCR Array.

**Results:**

PCR array analysis of 84 apoptosis-related genes demonstrated that 12 genes were up-regulated at least 4-fold and that one gene was down-regulated at least 2-fold in the *T. gondii* infection group compared with levels in the control group. The number of genes whose expression had increased during the period of infection with *T. gondii* was significantly higher than those whose expressions had decreased (18 versus 1) and Tnfrsf11b had the highest rate of gene expression.

**Conclusion:**

*T. gondii* induce *in vitro* apoptosis of GC-1 spg cells. This effect shows a trend of concentration-dependent increase so that with an increase in the ratio of parasite burden to spermatogonial cells, in addition to an increase in the number of genes whose expression has changed, the fold of these changes has increased as well.

## Introduction

Toxoplasmosis is caused by the protozoan *Toxoplasma gondii (T. gondii)*, obligate intracellular parasites that affect about 30% of the human population (1, 2). Drinking water, unwashed vegetables contaminated with cat feces containing oocytes, undercooked meat containing cysts, and receiving organs from an infected donor are the main routes of infection (3). Clonal strains of *T. gondii *are types I, II, and III (4, 5). Type I, the hypervirulent strain, causes infections leading to death in immunocompetent mice. Type II and III strains cause nonlethal infections due to their avirulent nature; the chronic latent infections are the main manifestations of these strains (6-8). Adults usually have no manifestations following infection with *T. gondii*; nevertheless, toxoplasmosis is associated with devastating outcomes in a developing fetus (9, 10). Apoptosis is a type of programmed cell death that is morphologically homogeneous (11, 12). Apoptosis morphologically shrinks nuclei and cytoplasms, condenses nuclear chromatin, dilates endoplasmic reticulum, and blebs membrane (13-15). Apoptosis is necessary in physiological conditions to regulate and fine-tune organelle function and architecture, it also can be induced or impeded during pathological conditions such as infections, inflammations, and cancer (16-19). 

A dynamic and synchronized maturation of stem spermatogonia into mature spermatozoa is called spermatogenesis; the testicular seminiferous tubules are the major site of spermatogenesis. the first wave of spermatogenesis occurs as soon as the gonocytes differentiate into spermatogonia; apoptosis increases also occur in this phase (20, 21). Some studies have investigated the rate of apoptosis in infertile males and shown interestingly the higher rate of apoptosis in the individuals (22-25). Moreover, they have also reported the percentage of apoptotic sperm is more abundant in ejaculated semen samples from infertile men. Therefore, it seems apoptosis can be regarded as one of the etiological molecular pathways involved in male infertility (26, 27). 

On the other hand, according to the reported evidence, there is a link between toxoplasmosis and the quality of human sperm (28). Decreased fertility was observed in rats infected with *T. gondii*, while the number of morphologically abnormal sperms increased, according to several studies (29). 

According to the results of several studies, toxoplasmosis also correlates with increased sperm apoptosis, especially in diploid spermatocytes (30), although the mechanism by which *T. gondii *alters reproductive parameters is still unknown.

Considering *T. gondii* correlation with opposing reports on the modulation of apoptosis induced by toxoplasmosis, the present study aimed at investigating the preventive mechanism(s) of apoptosis induction in *T. gondii*-infected spermatogonia cells in an *in vitro* system.

## Materials and Methods

***Ethics statement***

Animal care procedures in this study were performed according to International Guidelines for the Use of Laboratory Animals and ethical guidelines of the Animal Care Committee of Jundishapur University of Medical Sciences, Ahvaz, Iran. The Jundishapur University of Medical Sciences’ Ethics Committee also approved the procedures that were used in this study (code: IR.AJUMS.REC.1395.117). The animals’ health condition was checked via the experiments by a health surveillance program according to the Federation of European Laboratory Animal Science Associations (FELASA) guidelines. All efforts were made to minimize suffering.

***Experimental design; animal and tachyzoites***

Three BALB/c strain mice, 6–8 weeks old, weighing 20–22 g were used in the present study. The experimental animals were obtained from the Experimental Animal Unit of Razi Vaccine and Serum Research Institute, Iran. Mice were acclimatized to the laboratory conditions for 7 days prior to the initiation of experimental treatments. The experimental animals were housed in standard plastic cages and maintained under controlled laboratory conditions of humidity (55%), temperature (22–23 ^°^C), and 12:12 hr light:dark cycle. Mice were fed *ad libitum* normal commercial chow and had free access to water. 

The RH virulent strains of *T. gondii* were grown and maintained by routine intraperitoneal (IP) passage in BALB/c mice that were obtained from the Department of Parasitology and Mycology of Tehran University of Medical Sciences. In order to use fresh tachyzoites for exposuring with spermatogonia cells, firstly the fresh tachyzoites (2×10^2^) were injected IP to some mice, these mice were kept in a separate cage for 7 days until appearance of peritonitis and general weakness signs. On the 7^th^ day, the animals were euthanized using ketamine 10% (60 mg/kg) and xylazine 5% (10 mg/kg) according to standardized protocols supplied by the Jundishapur Medical Science Ethics Committee. The samples containing fresh tachyzoites were extracted from their peritoneal fluid in sterile conditions. The samples from infected mice were collected and washed twice (at 1,000×*g *for 15 min) in sterile phosphate-buffered saline (PBS; pH 7.2) then injected to other mice (2×10^2^ tachyzoites).

***Cell culture and treatment with T. gondii infection***

GC-1 spg cell line obtained from the Department of Cell Bank (Pasteur Institute of Iran) were used in this study. After preparing the cell line and confirmation of non-contamination of the medium, cell viability was measured using trypan blue 0.4% and then the cells were routinely counted manually with a hemocytometer. The cells were cultured in DMEM (Gibco, USA), supplemented with 10% (v/v) fetal bovine serum (Gibco, USA), 100 U/ml penicillin (Sigma-Aldrich, USA), and 100 μg/ml streptomycin (Sigma-Aldrich, USA). The cells were incubated at 37 °C in 5% CO_2_.

Tachyzoites were pelleted by centrifugation at 800×g for 10 min and resuspended in lysis buffer (150 mM NH_4_Cl, 0.1 M Tris–HCl pH 7.4) for 10 min at room temperature. Target cells were infected with *T. gondii* tachyzoites at a multiplicity of infection (MOI) of 1:1 in 100 μl of complete DMEM for 18 hr.

***Detection of apoptosis***

Cells were seeded in a six-well tissue culture dish. Designated wells were infected with ratio 1: 2 Tachyzoites/ GC-1 spg cell, passaged for 18 hr. According to the instructions, infected and noninfected cells were stained with annexin V using the Annexin V/PI kit (eBioscience™ Annexin V Apoptosis Detection Kit FITC) and apoptotic cells were identified and quantified by flow cytometry. Briefly, cells were washed in PBS and incubated with 10 X binding buffer, propidium iodide (PI), annexin V-FITC, and dH_2_O (total 100 μl) for 15 min in dark. Then stained cells were analyzed using a Faces Calibur flow cytometer (BD Biosciences, USA).

***Evaluation of genes associated with apoptosis process by RT*** ^2^ ***Profiler™ PCR Array***

Total RNA was isolated from target cells according to RNeasy Mini Kit (QIAGEN, Cat No. 74104) and the yield and quality of RNA were assessed by a spectrophotometer at 260 nm (Thermo Scientific NanoDrop™ One/OneC Microvolume UV Spectrophotometer, USA). 

Real-time PCR reactions were analyzed in total RNA using the Mouse Apoptosis RT^2^ Profiler™ PCR Array (QIAGEN, Cat No. PAMM-012ZA-24) according to the manufacturer’s protocol.

Briefly, cDNA was prepared from 500 ng total RNA using RT^2^ PCR array first strand kit (QIAGEN, Cat No. 330401). cDNA was diluted by adding RNase-free water. PCR was carried out using a LightCycler® 480 apparatus (Roche Applied Science). 

For one 96-well-plate of the PCR array, 2700 μl of PCR master mix (containing 1350 μl 2× RT^2^ SYBR Green Mastermix, 102 μl of cDNA synthesis reaction, and 1248 μl RNase-free water) were prepared, and aliquots of 25 μl were added to each well.

The relative gene expression was determined by QuantStudio 3, 96-well (Applied Biosystems™, USA) real-time detection system software using an adaptive baseline to determine the threshold cycle (CT). 

The data were analyzed by the ΔΔCT method according to the manufacturer’s manual. Quality control was performed using genomic DNA and reverse transcription and positive PCR controls. The data were normalized to the housekeeping genes. Changes in gene expression were represented as fold increase/decrease. The fold-change for each gene from group test to group control was calculated as 2^^-^^ΔΔct^. Genes were considered to be up-regulated or down-regulated if changes in expression levels were ≥ 1.0-fold or ≤ 1.0-fold, respectively.

***qPCR for candidate gene***

To validate the whole-genome microarray data, qRT-PCR was performed, therefore total RNA was extracted from two group cells (control and 2 Tachyzoites/cell) using a Super RNA extraction Kit (Yekta Tajhiz, Iran), and reverse transcription of the RNA was carried out using RevertAid First Strand cDNA Synthesis Kit (Thermo Scientific) according to the manufacturer’s instructions. cDNA was utilized as a template for subsequent qPCR amplification using primers specific for candidate genes. The qPCR reaction included an initial activation step at 94 ^°^C for 5 min, followed by 35 cycles at 94 ^°^C for 30 sec, annealing at 60 ^°^C for 30 sec, extension at 72 ^°^C for 30 sec, and a final extension at 72 ^°^C for 7 min. qPCR was performed using 5x HOT FIREPol ®EvaGreen ® qPCR Mix Plus, the real-time PCR kit (Solis BioDyne, Estonia), and Rotor-Gene Q Real-Time PCR System (Qiagen, USA). Raw data were obtained from Rotor-Gene Q Real-Time PCR System Software, exported in RDML format, and imported to LinRegPCR to calculate the PCR efﬁciency. Ct values generated in each experiment were used as an indicator to obtain fold change in the expression of target genes normalized to Gapdh. The relative expression levels in terms of fold change were calculated by the 2^-ΔΔCt^ method using the Gene software package (MultiD Analyses AB, Sweden). The primers used for cDNA amplification are shown in Table 1.

***Statistical analysis***

Gene’s expression were analyzed with RT^2^ PCR array data analysis version 3.4. Differences between groups were assessed by one-way analysis of variance (ANOVA). Differences were considered statistically significant at *P*<0.05. 

## Results

***Investigation of apoptosis***

**Figure 1:**
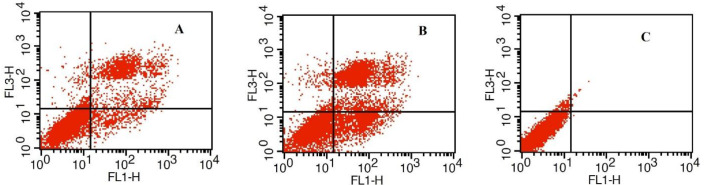
Flow cytometric analysis of apoptosis in mouse spermatogonia cells treated with *Toxoplasma gondii* for 18 hr. (%) indicates level of apoptosis

The percentage of apoptotic cells in group I (Tachyzoite / GC-1 spg cell) for 18 hr was 21.64 % cells; In group II (2 Tachyzoites / GC-1 spg cell) for 18 hr, the percentage of apoptotic cells was 43.69 % versus 1.34 % in the control group (untreated cells). the percentage of apoptotic cells in group II was significantly higher than group I and the control group (*P*<0.05) also there was significant different between group I and the control group (*P*<0.05) (Figure 1). 

***Expression changes of apoptosis-related genes***

**Figure 2:**
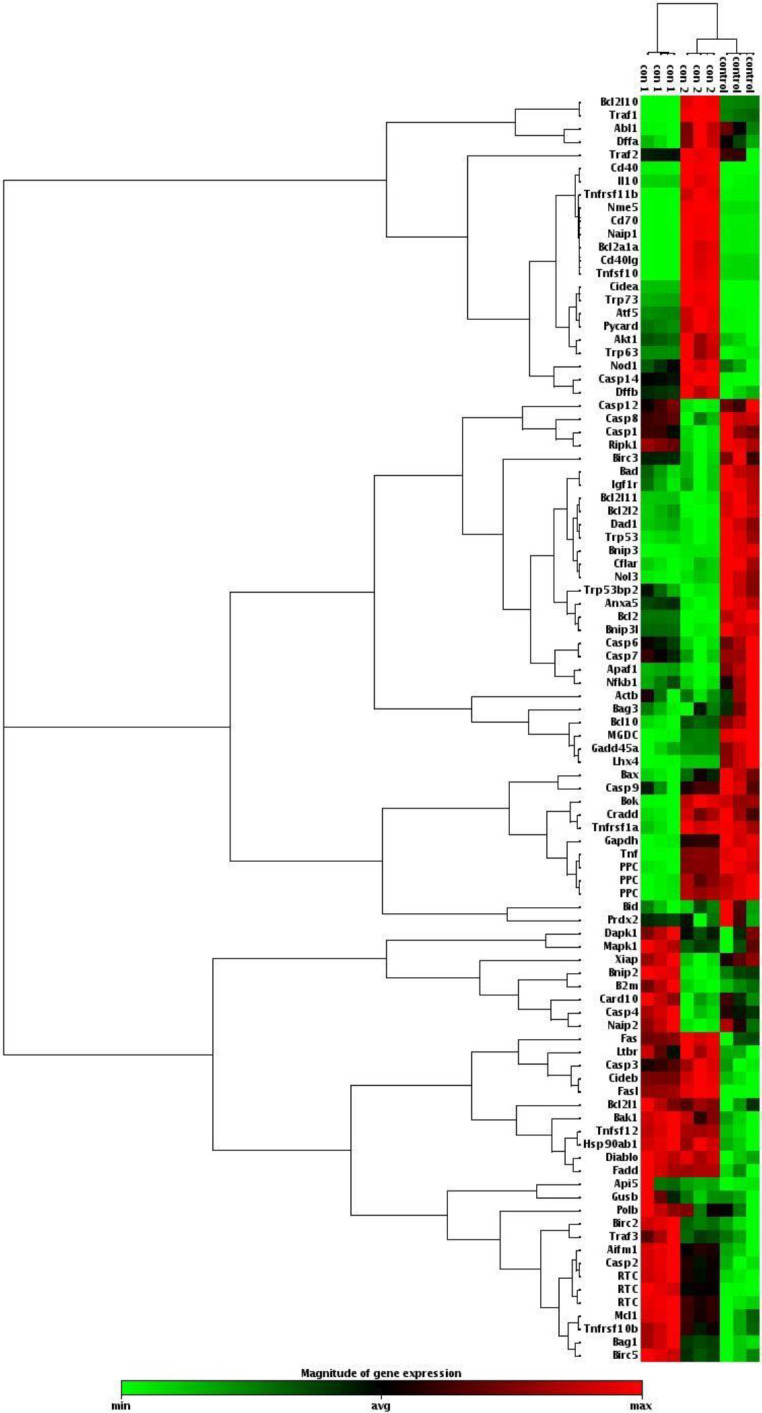
Cluster analysis of the down- and up-regulated apoptosis-related genes in GC – 1 spg cell line cells treated with different concentrations of *Toxoplasma gondii* tachyzoites (groups I and II) compared with the control group

To identify the potential effect of *T. gondii* tachyzoites on the cellular pathways of apoptosis-related genes of the GC-1 spg cell line, differences in the mRNA levels of selected genes were examined using a custom RT^2^ Profiler PCR array by comparing different concentrations of tachyzoites-treated cells with control (untreated) cells. Our results showed that group II had greater fold changes of apoptosis-related gene expression than group I compared with the control group. In both groups, we observed a significant down- and up-regulation of some genes. Four genes were more than 2-fold down-regulated in group I compared with the untreated group, but in group II, we showed the expression of a gene was decreased compared with the control group. In both treated groups, some genes showed increased expression. Especially, in group II, twelve genes were up-regulated more than 4-fold after treatment, with Tnfrsf11b presenting the most significant change (higher than 15-fold) (Table 2 and Figure 2). On the other hand, comparison between the two treated groups (I & II) showed that the expression of 14 genes in group II was significantly increased compared with group I (Table 2).

***Validation for candidate gene***

**Figure 3:**
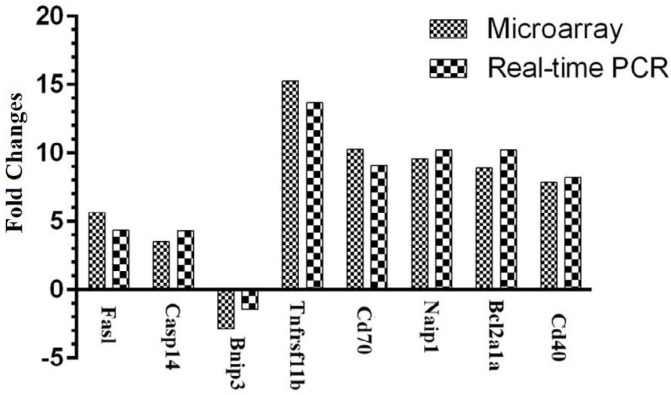
Concordance in the expression of the apoptosis genes between microarray and qRT-PCR

In order to confirm the accuracy of the results, qRT-PCR was carried out to validate the 10 genes. The results revealed that 8 of the 10 differently expressed genes were consistent with the data from microarray analysis. The fold changes of those 10 gene expressions, as determined by whole-genome microarray and qRT-PCR, are presented in Figure 3.

## Discussion

Whether *T. gondii* induces apoptosis or not is one of the questions that have long attracted the attention of researchers around the world. Various reports express contradictory results in this regard (40-48). Undoubtedly, unfolding the attractive and dual role of *T. gondii* in inducing or controlling apoptosis in the host cell, as well as its mechanism of action, would increase our understanding of the host-pathogen interaction mechanism of a significantly successful intracellular parasite.

By secreting virulence factors from its specialized organs, *T. gondii* can affect the gene expression modulation in host cells (49, 50). In general, some studies indicated the induction of high rates of apoptosis in different cell types, including splenocytes, T-cells in Peyer patches, and fibroblasts following the contamination of mice with *T. gondii* (48, 51, 52). However, some other studies showed that this parasite has no role in the induction of apoptosis in macrophages, neutrophils, lymphocytes B, and placental, spleen, and brain cells; this protozoan could even inhibit apoptosis through various mechanisms as reported in some studies (40, 53-57). In recent years, numerous studies demonstrated the negative effects of *Toxoplasma* on the reproductive system of humans and animals, especially females (58-61). Moreover, a few studies indicated that this parasite is a role-player in the infertility of male patients (62). Therefore, this study aimed at investigating the effects of *T. gondii* on changing the expression of apoptosis-related genes in B-type spermatogonial cells.

In spermatogonial cells, apoptosis usually takes place to prevent the excessive production of germ cells and eliminate the damaged germ cells. However, infectious agents, smoking, radiation, pesticides, etc., may also induce pathological apoptosis in these cells (11, 12, 63). The present study showed that adjoining RH strain tachyzoites of *T. gondii* with B-type spermatogonial cells with a 2:1 ratio significantly induced apoptosis when compared with the control group. However, at lower ratios, no significant differences were observed between the two groups in terms of the incidence of apoptosis, which may be indicative of the dose-dependent nature of apoptosis induced by *T. gondii*.

*T. gondii* strains with different virulent factors might be some of the causes of the discrepancy between previous studies regarding the induction of apoptosis by this parasite (51). Overall, *T.* *gondii* strains are categorized into three groups: I, II, and III, based on their virulence to mice; each of which uses different mechanisms to stimulate or destroy the host immune response (64, 65). A study examined the virulence role of various strains of *T. gondii* in the host immune response. It showed that the strain with high virulence (RH) proliferated and multiplied faster than that with less virulence (ME49) and was more fatal in mice (51). In addition, nitric oxide and IFN-γ levels increased in serum and peritoneal fluid of mice infected with high-virulent strains; whereas such increases were not observed in mice infected with low-virulent strains (66).

Acute or chronic nature of infection can affect the pathogenesis of toxoplasmosis, according to the findings of some studies. Two researchers reported that the contamination of human monocytes with *T. gondii* in the acute phase results in the release of soluble factors (67). These factors could lead to apoptosis induction and immune system weakening. The evidence indicates that non-contaminated cells may also suffer from *T. gondii* under the influence of NO and other soluble factors released from the infected cells. According to the results of a study, in acute infections, non-contaminated host cells could act as spectators, and excessive production of Th1 cytokines could greatly contribute to pathogenesis (66). Some other studies showed that during acute toxoplasmosis, IFN-γ can result in apoptosis and suppression of the immune system (51, 52, 68).

*In vitro* conditions of previous studies could be one of the reasons for the dual behavior of *T. gondii* in inducing or inhibiting apoptosis (69). The duration of infection in a target cell with the parasite is another issue to be considered. *T. gondii* may in regulated to proliferation and surviving itself, playing an opposite role in apoptosis at different times (70).

Nishikawa *et al*. comparing *in vitro* and *in vivo* methods, showed no significant difference between the two strains with high and low virulence in the induction of apoptosis in cells *in vitro*, whereas the more virulent strain significantly induced higher rates of apoptosis *in vivo* (66). Due to the presence of different cell types in the environment and their interaction with each other, the situation is more complicated *in vivo*. 

In a study by Nash *et al*. human fibroblasts were used to maintain *T. gondii* tachyzoites (41), but in the present study, newly acquired tachyzoites in the peritoneal fluid from BALB/c mice were used for testing. These different conditions could justify the contradictory results obtained in the two studies.

Among the key issues, employing different target cells in previous studies is noteworthy. For instance, the study by Young Hwang *et al*. showed that infection caused by *T. gondii* prevented the incidence of apoptosis in a human leukemia cell line (THP-1) (71). However, the study by Nishikawa *et al*. showed that *T. gondii* induced apoptosis in mice fibroblasts (BALB/3T3 clone A31 fibroblasts) (48). As the biological characteristics of these cells are different, it is quite normal that the responses of these cells also vary in the presence of *T. gondii*.

Two known pathways can result in apoptotic cell death. One of them is through the transfusion of receptors with extracellular intermediates (TNF-α/TNFR I; Fas/FasL) (72), and the other is through mitochondrial oxidation (73, 74). It is reported that a number of factors, such as TNF-α, Fas, NF-κB, and p53, are involved in the regulation of apoptosis pathways. However, according to evidence, *T. gondii* can significantly modulate host cell-related transcription factors (49).

In the present study, the comparison between the 2X and control groups indicated that *T. gondii* led to increased expression of several pro- and anti-apoptotic genes and only reduced the expression of one anti-apoptotic gene. The highest increase in expression (15.28 times) was related to Tnfrsf11b. In addition, the expression of Fasl, Cidea, Cd70, Naip1, Bcl2a1a, Cd40, Cd40lg, Trp73, Il10, Tnfsf10, and Nme5 genes increased more than four times. The expression of pro-apoptotic genes induced apoptosis in three different pathways including TNF-α family (Tnfrsf11b, Cd70, Cd40, and Tnfsf10), p53 pathway (Trp73), and the CIDE (Cidea) family; whereas the increased expression of anti-apoptotic genes including TNF-α (Cd40lg), IAP (Naip1), NME/NM23 (Nme5), Bcl2 (Bcl2a1a), and Il10 families was involved in the inhibition of apoptosis. The induction of apoptosis in the present study might be justified as changes in the expression of genes with positive effects on the apoptosis pathway were greater than those of genes with negative effects. Moreover, the number of genes whose expression increased during infection with *T. gondii* was significantly higher than that of those whose expression decreased (18 versus 1), indicating that the induction of gene expression might be the most common response of type B spermatogonia cells to infection.

As mentioned earlier, the induction of apoptosis in type B spermatogonia cells by *T. gondii* is dose-dependent so that with an increase in the ratio of parasite mass to spermatogonial cells, in addition to an increase in the number of genes whose expression changed, the fold of these changes increased, as well.

**Table 1:** Primer sequences of candidate gene use to validation of RT2 Profiler™ PCR methods

Gene symbol	Primer sequences	
fasl (31)	forward	CGGTGGTATTTTTCATGGTTCTGG
	reverse	CTTGTGGTTTAGGGGCTGGTTGTT
casp14	forward	ATGAGCAATCCGCGGTCTTTGG
	reverse	CTGCAGATACAGCCGGAG
bcl2l10 (32)	forward	GCCAACCTTTGTTCATGGC
	reverse	GTGGTGACGCTCGTGACC
bnip3 (33)	forward	GTTCCAGCCTCGGTTTCTA
	reverse	TAGAAACCGAGGCTGGAAC
tnfrsf11b (34)	forward	GTTTACTTTGGTGCCAGG
	reverse	GCTTGAAACATAGGAGCTG
cd70 (35)	forward	AGCGGACTACTCAGTAAGCAGCAAC
	reverse	CAGCTCTGGTCCGTGTGTGAA
naip1 (36)	forward	GGGACATCACCACGTGTACTC
	reverse	TTGTTGTGCTCTTGTATTGGG
bcl2a1a (37)	forward	GATACGGCAGAATGGAGGTT
	reverse	GCATTTCCCAGATCTGTCCT
cd40 (38)	forward	GGAGATGGAAGATTATCCCGG
	reverse	GGCATGAGAGTTAGCTGCAC
trp73 (39)	forward	CTGGTCCAGGAGGTGAGACTGAGGC
	reverse	CTGGCCCTCTCAGCTTGTGCCACTTC
gapdh (35)	forward	AAATGGTGAAGGTCGGTGTG
	reverse	TGAAGGGGTCGTTGATGG

(A) group I (Tachyzoite / GC-1 spg cell), 21.64%; (B) group II (2 Tachyzoites / GC-1 spg cell), 43.69%; (C) control group (untreated cells) for 18 hr incubation. Flow cytometry was repeated more than three times and similar results were observed

**Table 2:** Relative fold changes of apoptosis- related genes expression in group I , II and control group of GC-1 spg cell

Gene symbol (Full Name)	(group I /control)	(group II /control)	(group II / group I)
fasl (fas ligand)	4.80	5.66	unchanged
casp14 (caspase 14)	2.23	3.51	unchanged
cidea (cell death-inducing DNA fragmentation factor)	2.15	10.27	4.78
tnf (tumor necrosis factor)	-9.75	unchanged	7.93
bnip3 (baculoviral IAP repeat-containing 3)	-3.11	-2.86	Unchanged
traf1 (tnf receptor-associated factor 1)	-2.33	2.50	5.81
bcl2l10 (bcl2-like 10)	-2.26	2.68	6.05
tnfrsf11b (tumor necrosis factor receptor superfamily, member 11b)	unchanged	15.28	22.13
cd70 (cd70 antigen)	unchanged	10.27	14.40
naip1 (NLR family, apoptosis inhibitory protein 1)	unchanged	9.58	13.44
bcl2a1a (b-cell leukemia/lymphoma 2 related protein A1a)	unchanged	8.88	12.45
cd40 (cd40 antigen)	unchanged	7.84	6.95
cd40lg (cd40 ligand)	unchanged	4.56	6.40
trp73 (transformation related protein 73)	unchanged	5.74	3.20
l10 (interleukin 10)	unchanged	5.43	4.05
tnfsf10 (tumor necrosis factor (ligand) superfamily, member 10)	unchanged	4.53	6.36
nme5 (non-metastatic cells 5)	unchanged	5.82	8.16
trp63 (transformation related protein 63)	unchanged	2.64	unchanged
atf5 (activating transcription factor 5)	unchanged	2.21	unchanged
cideb (cell death-inducing DNA fragmentation factor, alpha subunit-like effector B)	unchanged	2.13	unchanged

Expression levels above the mean for the gene are shown in red squares and expression

levels below the mean for the gene are shown in green squares

## Conclusion

*T. gondii* induces apoptosis in spermatogonial cells directly or indirectly (by releasing soluble factors from infected cells). Many factors, including parasite virulence, duration of infection, parasite mass, and target cell type, may affect this process. The limitations of the present study included: first, the lack of elevation of the expression of proteins whose genes were evaluated; secondly, the unavailability of two strains with different actuations simultaneously.

## Declaration

***Ethics approval and consent to participate***

This clinical protocol was approved by the Research Ethics Committee of Ahvaz Jundishapur University of Medical Sciences (protocol number IRAJUMS.REC.1395. 117). 
